# Supplementary material for: Occurrence records and metadata for sand flies (Diptera, Psychodidae, Phlebotominae) collected in the lands of indigenous people in the Brazilian Amazon
Source: GigaByte. 2022 May 31;2022:gigabyte61. doi: 10.46471/gigabyte.61 (PMC9876538; doi:10.46471/gigabyte.61)
Supplement: Translation

# Translation: Registros de ocorrência e metadados de flebotomíneos (Diptera, Psychodidae, Phlebotominae) coletados em terras indígenas na Amazônia brasileira

**Authors:** Paloma Helena Fernandes Shimabukuro, Daniel Rocha Cangussu Alves, Jéssica Adalia Costa Barros, Luiz Otavio Cordeiro Nascimento, Luke Anthony Baton, Maíra Posteraro Freire, Manoel Edson Medeiros da Silva, Mauro Diego Gobira Guimarães de Assis, Sofia Ferreira Morais, Tiago Silva da Costa, Veracilda Ribeiro Alves, Eduardo Stramandinoli Moreno

**Affiliations:** ^1^ Coleção de Flebotomíneos (FIOCRUZ/COLFLEB), Fiocruz, Belo Horizonte, Brazil; ^2^ Grupo de Estudos em Leishmanioses, Fiocruz, Belo Horizonte, Brazil; ^3^ Frente de Proteção Etnoambiental Médio Purus/Coordenação-Geral de Índios Isolados e de Recente Contato – CGIIRC/Fundação Nacional do Índio. Lábrea, Amazonas, Brazil; ^4^ Distrito Sanitário Especial Indígena Amapá e Norte do Pará – Secretaria Especial de Saúde Indígena - Ministério da Saúde, Macapá, Amapá, Brazil; ^5^ 50 Rowntree Way, Saffron Walden, Essex CB11 4DL, UK; ^6^ Programa de Pós-Graduação em Saúde, Sociedade e Endemias na Amazônia, Instituto Leônidas e Maria Deane, Fundação Oswaldo Cruz/Universidade Federal do Amazonas – Manaus, Amazonas, Brazil; ^7^ Laboratório de Arthropoda (Arthrolab), Universidade Federal do Amapá (Unifap), Macapá, Amapá, Brazil; ^8^ Instituto Evandro Chagas, Ananindeua, Pará, Brazil; ^9^ Programa de Pós-Graduação: Sociedade, Natureza e Desenvolvimento – Universidade Federal do Oeste do Pará, Santarém, Pará, Brazil

## Abstract

Para contribuir com o conhecimento da epidemiologia da leishmaniose tegumentar americana (LTA) entre indígenas que vivem em regiões silvestres, estudamos a fauna de flebotomíneos coletada em áreas de transmissão da doença na Amazônia brasileira. Aqui relatamos dois conjuntos de dados que incluem dados de ocorrência de flebotomíneos da Terra Indígena Suruwaha no estado do Amazonas coletados em 2012–2013 e da Terra Indígena Wajãpi no estado do Amapá coletados em 2013–2014. Os flebotomíneos foram coletados usando armadilhas de luz tipo CDC sem isca em vários locais dentro de cada área de estudo e foram identificados em nível de gênero ou espécie por taxonomistas com experiência em fauna amazônica. São registrados 4.646 registros: 1.428 dos Suruwaha e 3.218 dos Wajãpi. Esses registros contribuirão para um melhor entendimento da dinâmica de transmissão do LTA, bem como da distribuição de insetos vetores, nessas áreas.

## Descrição dos Dados

As leishmanioses são doenças causadas por várias espécies do protozoário parasita do gênero *Leishmania*, que são transmitidas entre humanos, animais vertebrados selvagens e domésticos, pela picada de flebotomíneos fêmeas que se alimentam de sangue [1.]. No Brasil, a leishmaniose tegumentar americana (LTA) é uma doença endêmica, mas pouco se sabe sobre seu impacto nas populações humanas indígenas, principalmente aquelas que vivem em áreas remotas e têm pouco contato com não indígenas que vivem fora de suas terras. Para investigar a fauna de flebotomíneos presentes durante os surtos de LTA entre os indígenas Suruwaha e Wajãpi ocorridos entre 2012 e 2014, realizamos trabalho de campo para coletar esses insetos e identificar possíveis vetores. Os insetos foram identificados por taxonomistas experientes usando chaves disponíveis na literatura [2., 3.].

Nossos conjuntos de dados compreendem dados de ocorrência de flebotomíneos da: (i) Terra Indígena Suruwaha (TIS) no sul do estado do Amazonas coletados entre 2012 e 2013; e (ii) a Terra Indígena Wajãpi (TIW) no estado do Amapá coletada entre 2013 e 2014. Os conjuntos de dados aqui relatados são os metadados para cada espécime individual de flebotomíneos coletado durante o trabalho de campo e incluem 41 termos Darwin Core Standard (DwC) [4.] para o conjunto de dados Suruwaha e 39 para o conjunto de dados Wajãpi. Todos os campos obrigatórios estão presentes e passaram por triagem no kit de ferramentas de publicação integrada (IPT) da Fundação Oswaldo Cruz (FIOCRUZ). Os campos de metadados também estão disponíveis nas páginas online [5., 6.].

Para cada espécime individual de flebotomíneos, nosso conjunto de dados inclui campos que descrevem: (i) taxonomia (kingdom, phylum, class, order, family, genus, specificEpithet, verbatimIdentification, infraspecificEpithet, scientificName, scientificNameAuthorship, taxonRank); (ii) detalhes da coleção, incluindo os coletores (recordedBy: Shimabukuro PHF; Stumpp RGAV; Medeiros MES.; Alves DRC; Moreno ES; Freire M P; Nascimento LOC), data de coleta, método de captura, número de identificação da armadilha e descrição do local de coleta (verbatimEventDate, eventTime, habitat, samplingProtocol); (iii) dados de geolocalização (stateProvince, county, locality, locationRemarks, verbatimLatitude, verbatimLongitude, decimalLatitude, decimalLongitude, geodeticDatum); e (iv) dados de referência do catálogo (otherCatalogNumbers).

Nossos conjuntos de dados [5., 6.] estão disponíveis publicamente no Sistema de Informação sobre a Biodiversidade Brasileira (SiBBr). Esta é uma plataforma online que integra dados e informações sobre biodiversidade e ecossistemas e é o Nó Brasileiro do Global Biodiversity Information Facility (GBIF), um recurso internacionalmente reconhecido para compilação de dados de ocorrência biológica [7.].

### Contexto

A LTA é um grave problema de saúde pública no Brasil, com 203.406 casos registrados entre 2010 e 2020. A Região Administrativa no Norte do país, que inclui o bioma Amazônia, atualmente responde por 42% de todos os casos registrados no Brasil [8.].

As condições de saúde e doença entre os povos indígenas no Brasil são pouco compreendidas. As bases de dados governamentais apresentam grandes lacunas nas informações que possuem sobre morbidade, mortalidade, notificação de doenças entre indígenas e assim por diante, o que impede a construção dos indicadores sociodemográficos e de saúde mais básicos. No entanto, os poucos estudos sobre a saúde pública das populações indígenas no Brasil revelam marginalização e vulnerabilidade, que se traduzem em baixa qualidade de vida, dificuldade de acesso aos serviços e baixos indicadores de saúde [9.].

Os diferentes padrões de transmissão da LTA também a tornam uma doença difícil de controlar e entender. Isso é especialmente verdade na Amazônia brasileira, que possui a maior diversidade de parasitas de espécies de *Leishmania*, seus insetos vetores e hospedeiros vertebrados, na região neotropical [1.].

Nossos dois conjuntos de dados incluíram dados de ocorrência de flebotomíneos do trabalho de campo realizado pelos autores durante os surtos de ACL no TIS e TIW.

### Conjunto de dados Suruwaha

A transmissão de ACL ao sul do sistema do rio Amazonas é pouco compreendida [10., 11.]. Embora muitas vezes tenha sido afirmado que a infecção humana por *Leishmania* é rara ou ausente ao sul dos rios Negro e Amazonas [10., 12.], a incidência de leishmaniose em humanos em algumas dessas áreas é equivalente à incidência ao norte dos rios Negro e Amazonas [11., 13.]. Guerra *et al.* [14.] descreveram a epidemiologia da leishmaniose mucosa (LM) ao sul do rio Amazonas, e encontraram não apenas uma alta prevalência dessa forma da doença, mas também uma distribuição de espécies de *Leishmania* semelhante à encontrada ao norte da Rio Amazonas. No entanto, o agente etiológico da leishmaniose tegumentar (LC) não foi identificado, e a fonte de infecção silvestre e os vetores envolvidos na transmissão não são conhecidos ao sul do sistema do rio Amazonas [10., 15.].

Os Suruwaha são uma população de aproximadamente 170 pessoas [16.], cujo único contato com não indígenas se dá por meio de agentes de saúde e membros da Fundação Nacional do Índio (FUNAI), órgão do governo brasileiro responsável pela proteção dos povos indígenas. Os Suruwaha vivem em local remoto, longe das áreas urbanas e da fabricação de utensílios. Suas principais atividades são a agricultura e a caça.

Realizamos pesquisas entomológicas dentro da TIS para contribuir para o entendimento da dinâmica de transmissão da LTA. Esta pesquisa foi uma resposta ao aumento no número de casos de LTA registrados entre os Suruwaha entre 2010 e 2012. Nossa investigação constatou que esse aumento provavelmente estava relacionado à distribuição de lanternas para toda a comunidade ao final de 2010, conforme solicitado pelos Suruwaha mesmos. O uso dessas lanternas levou a mudanças nos hábitos de caça dos Suruwaha, de estritamente diurnos para noturnos, coincidindo com a atividade de picada geralmente crepuscular e noturna das fêmeas hematófagas que transmitem LTA.

A TIS tem localização remota. Nosso levantamento entomológico foi realizado por diferentes equipes para aproveitar as operações rotineiras da FUNAI. Um total de 1.428 espécimes de flebotomíneos foram identificados em nível de gênero ou espécie. Alguns espécimes não puderam ser identificados ao nível de espécie porque são morfologicamente indistinguíveis, as estruturas morfológicas não ficaram claramente visíveis na lâmina ou os espécimes perderam apêndices importantes para a identificação morfológica. Foram coletados dez gêneros e 33 espécies (Tabela 1), sendo o gênero *Trichophoromyia* predominante (57%), seguido de *Psychodopygus* (20%) e *Nyssomyia* (5%). Entre as espécies mais abundantes estavam *T. ubiquitalis* (25%), *T. octavioi* (9%) e *Ps. davisi* (8%). Um total de 667 espécimes femininos e 760 masculinos foram coletados. Um espécime perdeu a cabeça e a terminália, portanto não pôde ser sexado.

**Tabela 1:** Espécies de flebotomíneos coletadas na Terra Indígena Suruwaha, município de Tapauá, estado do Amazonas, entre 2012 e 2013.

Species	Number of specimens
*Trichophoromyia ubiquitalis*	634
*Trichophoromyia* sp.	146
*Trichophoromyia octavioi*	133
*Psychodopygus davisi*	123
*Psychodopygus chagasi*	80
*Psychodopygus claustrei*	42
*Bichromomyia flaviscutellata*	42
*Trichophoromyia melloi*	41
*Nyssomyia antunesi*	33
*Psychodopygus ayrozai*	23
*Nyssomyia fraihai*	22
*Psychodopygus* sp.	13
*Nyssomyia* sp.	10
*Psychodopygus paraensis*	10
*Psathyromyia* sp.	7
*Psychodopygus geniculatus*	6
*Sciopemyia sordellii*	6
*Evandromyia saulensis*	5
*Evandromyia* sp.	6
*Lutzomyia* sp.	6
*Sciopemyia* sp.	4
*Trichophoromyia brachipyga*	4
*Psathyromyia barrettoi barrettoi*	4
*Evandromyia bacula*	3
*Evandromyia begonae*	3
*Lutzomyia evangelistai*	3
*Psathyromyia abunaensis*	2
*Psychodopygus hirsutus*	2
*Psychodopygus llanosmartinsi*	2
*Psychodopygus amazonensis*	1
*Psathyromyia aragaoi*	1
*Viannamyia furcata*	1
*Psathyromyia inflata*	1
*Psychodopygus lainsoni*	1
*Bichromomyia olmeca nociva*	1
*Bichromomyia olmeca reducta*	1
*Psathyromyia runoides*	1
*Sciopemyia servulolimai*	1
*Viannamyia tuberculata*	1
*Trichopygomyia* sp.	1
*Trichopygomyia wagleyi*	1
Total	1428

### Conjunto de dados Wajãpi

Os Wajãpi são um grupo de cerca de 1.200 pessoas [17.], distribuídas em 80 pequenas aldeias em todo o seu território. Todas as famílias subsistem da agricultura, pesca, caça e coleta, mudando periodicamente a localização de suas aldeias para permitir a recuperação ecológica das áreas que ocuparam. O acesso às aldeias pode ser feito por estrada, rios e córregos que atravessam seu território, e trilhas abertas pela mata. A retomada do modelo tradicional de ocupação e dispersão wajãpi para exploração territorial, que mantém a qualidade de vida do povo e, ao mesmo tempo, garante a vigilância territorial, foi essencial para garantir o padrão de abundância que os Wajãpi consideram adequado [18.]. Nos últimos 10 anos, a atividade de grandes mineradoras e a construção de hidrelétricas no entorno do TIW ameaça a qualidade de vida dos Wajãpi por meio da degradação florestal, contaminação ambiental e inserção da comunidade em um contexto de grande vulnerabilidade social, coincidindo com o aumento da incidência de pragas nas lavouras e doenças endêmicas, como malária e leishmaniose.

Nosso conjunto de dados compreende registros de flebotomíneos resultantes das atividades de um projeto de avaliação dos principais fatores de risco para a doença, utilizando uma abordagem interdisciplinar. Em 2012, houve um surto de leishmaniose na TIW afetando mais de 20 pessoas em sete aldeias diferentes (dados fornecidos pela SESAI). Esse surto desencadeou uma investigação mais ampla que foi realizada entre 2012 e 2015 para compreender os processos causais relacionados ao contexto epidemiológico local, para propor medidas de prevenção e controle da doença entre os Wajãpi. A investigação desdobrou-se em um projeto multiinstitucional com a parceria de instituições ligadas aos serviços de saúde, tais como: Distrito Sanitário Especial Indígena (DSEI) Amapá e Norte do Pará, Secretaria Municipal de Saúde de Pedra Branca do Amapari, Coordenadoria de Vigilância em Saúde do Amapá, Laboratório Central de Saúde Pública do Amapá, Instituto René Rachou (Fiocruz-MG), Universidade Federal do Amapá, Universidade de São Paulo, e Universidade Federal do Oeste do Pará. Formaram-se equipes com diferentes especialidades: entomologistas, biólogos, veterinários, técnicos de laboratório, antropólogos, epidemiologistas, enfermeiros, etc.

Desde o início, os pesquisadores se depararam com um contexto ambiental e intercultural complexo, no qual a simples intensificação das medidas preconizadas pelos serviços de saúde se mostrou insuficiente – ou mesmo antagônica em relação aos princípios e conceitos de saúde do grupo indígena. O difícil acesso às aldeias dificultou o mapeamento e registro de possíveis locais de transmissão. O longo período de incubação do ACL, o complexo perfil de mobilidade pelo território dos Wajãpi e as limitações dos serviços de saúde, por exemplo, falta de pessoal capacitado, falta de financiamento e infraestrutura adequados, fizeram com que a busca por focos de transmissão, segundo os padrões de vigilância preconizados pelo Ministério da Saúde – baseados na busca de fatores de risco individuais, tarefa pouco promissora.

A necessidade de uma avaliação mais ampla e sensível do ponto de vista ecológico, cultural e étnico desse contexto epidemiológico se desdobrou em uma revisão – não apenas dos objetivos e métodos de prestação de serviços em saúde indígena e de pesquisas e condutas epidemiológicas, mas também de uma reconstrução epistemológica do processo de compreensão da causalidade na saúde-doença e sua relação indissociável com os processos sociais e ecológicos, suas diferentes escalas e dimensões de percepção. Portanto, os dados sobre a ocorrência de vetores ACL foram primordiais.

Um total de 3.218 espécimes (Tabela 2) foram identificados em nível de gênero ou espécie. Novamente, algumas espécies não puderam ser identificadas ao nível de espécie, pois eram morfologicamente indistinguíveis, as estruturas morfológicas não eram claramente visíveis na lâmina ou os espécimes perderam apêndices importantes para a identificação morfológica. Os gêneros mais abundantes foram *Trichophoromyia* (20%), *Nyssomyia* (13%) e *Psathyromyia* (11%). As espécies mais abundantes foram *T. brachipyga* (14%), seguida de *Pa. dreisbachi* (9%) e *Nyssomyia pajoti* (6%). Um total de 1.938 espécimes femininos e 1.276 masculinos foram coletados. Quatro espécimes perderam cabeça e terminália e não puderam ser sexados.

**Tabela 2:** Espécies de flebotomíneos coletadas na Terra Indígena Wajãpi, município de Pedra Branca do Amapari, estado do Amazonas, entre 2013 e 2014.

Species	Number of specimens
*Trichophoromyia brachipyga*	456
*Trichophoromyia* sp.	345
*Psathyromyia dreisbachi*	298
*Nyssomyia pajoti*	204
*Pressatia* sp.	198
*Evandromyia infraspinosa*	195
*Trichophoromyia ubiquitalis*	173
*Nyssomyia umbratilis*	169
*Trichopygomyia trichopyga*	165
*Bichromomyia flaviscutellata*	128
*Psychodopygus davisi*	112
*Sciopemyia sordellii*	110
*Evandromyia brachyphalla*	78
*Trichopygomyia* sp.	71
*Evandromyia* sp.	53
*Psychodopygus* sp.	47
*Psychodopygus geniculatus*	33
*Evandromyia monstruosa*	33
*Nyssomyia antunesi*	31
*Psathyromyia inflata*	29
*Nyssomyia* sp.	26
*Psychodopygus hirsutus*	22
*Psathyromyia* sp.	16
*Evandromyia andersoni*	15
*Psathyromyia aragaoi*	14
*Viannamyia furcata*	12
*Psychodopygus paraensis*	12
*Micropygomyia rorotaensis*	12
*Psychodopygus claustrei*	11
*Psychodopygus maripaensis*	11
*Migonemyia migonei*	10
*Nyssomyia whitmani*	9
*Micropygomyia* sp.	8
*Evandromyia pinottii*	8
*Viannamyia tuberculata*	8
*Pintomyia damascenoi*	7
*Sciopemyia fluviatilis*	7
*Brumptomyia pentacantha*	7
*Brumptomyia* sp.	6
*Psychodopygus corossoniensis*	6
*Sciopemyia* sp.	5
*Pressatia choti*	5
*Psathyromyia dendrophyla*	5
*Trichopygomyia depaquiti*	5
*Trichophoromyia ininii*	5
*Bichromomyia* sp.	4
*Psathyromyia barrettoi barrettoi*	3
*Psathyromyia lutziana*	3
*Psathyromyia pradobarrentosi*	3
*Lutzomyia sherlocki*	3
*Lutzomyia* sp.	2
*Psathyromyia abunaensis*	2
*Pressatia trispinosa*	2
*Psathyomyia abonnenci*	1
*Psychodopygus amazonensis*	1
*Nyssomyia anduzei*	1
*Evandromyia* sp. de Baduel	1
*Evandromyia begonae*	1
*Psathyromyia bigeniculada*	1
*Brumptomyia brumpti*	1
*Migonemyia bursiformis*	1
*Lutzomyia gomezi*	1
*Sciopemyia nematoducta*	1
*Pintomyia pacae*	1
*Psathyromyia runoides*	1
*Psathyromyia scaffi*	1
*Pintomyia serrana*	1
*Sciopemyia servulolimai*	1
*Micropygomyia trinidadensis*	1
Total	3,218

## Métodos

As amostras foram georreferenciadas com o auxílio de um dispositivo de sistema de posicionamento global (GPS) Garmin^®^ 62s.

### Área de estudo

#### Terra Indígena Suruwaha (TIS)

O município de Tapauá está localizado às margens do Rio Purus. Tem uma população de 16.876 habitantes e uma área total de 84.946 km^2^ [13.]. A principal atividade econômica é a agricultura (mandioca, juta e feijão) e o extrativismo (castanha, borracha, madeira, óleo de copaíba e andiroba). A pecuária se tornou o principal produto nos últimos 10 anos.

A região do Médio Purus fica no estado do Amazonas, na parte sul da floresta amazônica. Inclui diversas unidades de conservação, além de diversas terras indígenas, várias das quais já reconhecidas oficialmente pelo governo federal, enquanto outras estão em processo de demarcação legal formal.

De acordo com o SIASI (Sistema de Informação da Atenção à Saúde Indígena), operado pela Secretaria Especial de Saúde Indígena (SESAI), a população indígena do Médio Purus é estimada em cerca de 8.117 habitantes. Estas, estão divididas em 13 etnias e distribuídas em 24 terras indígenas demarcadas e outras 6 não demarcadas (não identificadas e/ou delimitadas). A extensão territorial total das áreas indígenas demarcadas corresponde a 189.870,964 ha.

A Terra Indígena Suruwaha (TIS) fica no município de Tapauá. A área do SIL compreende 239.070 ha e está localizada entre o rio Riozinho e o córrego Coxodoá, ambos afluentes do rio Cuniuá, que por sua vez é um afluente do rio Tapauá que deságua no rio Purus [19.]. A TIS é cercado a oeste pela Terra Indígena Deni, e a leste pela Terra Indígena Hi-Merimã, de povos indígenas isolados. A população é composta por 171 pessoas que viviam isoladas e foram contatadas por missionários na década de 1980 [20.]. As atividades produtivas dos Suruwaha incluem agricultura, caça, pesca, coleta e fabricação de ferramentas [19.]. A caça é a atividade de maior prestígio, e um bom caçador não só mata muitos animais, mas também deve ter matado muitas antas, a caça mais cobiçada pelo seu tamanho, que pode alimentar muitas pessoas [20.].

A LTA é endêmica da região amazônica, e o estado do Amazonas contribuiu com 20% (34.169 casos) do total de casos (167.186) registrados para a região norte do Brasil, onde está localizada a Amazônia, entre 2002 e 2020 [5.]. Veja na Figura 1 o mapa das ocorrências georreferenciadas no GBIF.

**Figura 1.** Mapa interativo das ocorrências georreferenciadas de flebotomíneos da Terra Indígena Suruwaha, hospedado pelo GBIF [21.]. https://www.gbif.org/dataset/d9f6d2bf-20d7-4b87-99ac-e902b34364e4

#### Terra Indígena Wajãpi (TIW)

A Terra Indígena Wajãpi (TIW) se estende entre as bacias dos rios Jari (a oeste), Amapari (a leste) e Oiapoque (ao norte). O reconhecimento oficial pelo governo brasileiro ocorreu em 1996, com uma extensão territorial de 6.070 km^2^. A área é de floresta tropical densa e de relevo acidentado, integrando o complexo da Serra do Tumucumaque. Atualmente, os Wajãpi somam cerca de 1.221 pessoas, distribuídas em mais de 80 aldeias [17.].

Os Wajãpi se organizam em grupos locais autônomos denominados ‘iwanã-ko’, que são representados por um grupo local que tem sua origem em uma região específica, onde existem várias aldeias. No entanto, nem todas as pessoas de um iwanã-ko vivem na mesma região, pois quando ocorrem casamentos entre pessoas de grupos diferentes, um dos cônjuges passa a residir na região do outro [22.], o que pode representar apenas um assentamento físico temporário. Os diferentes grupos ocupam três categorias espaciais: os locais de concentração: aldeias/moradias rurais; dispersão/assentamentos intermitentes (principalmente durante a estação seca – acampamentos de caça, pesca e coleta); e sítios ‘koo kwerã’, que são reservas de vida selvagem deixadas intactas para uso de animais selvagens e, portanto, serem caçados. Essa combinação de fatores sociais, políticos e ecológicos que determinam os movimentos de concentração e dispersão dos Wajãpi em seu território parece acompanhar os padrões de distribuição da doença na TIW, de modo que a tendência de concentração de famílias próximas à rodovia Perimetral Norte, que atinge a TIW, parece estar relacionada ao aumento da incidência de doenças infecciosas [18., 23.].

O estado do Amapá contribuiu com 6% (10.125) do total de casos (167.186) de LTA registrados para a região norte do Brasil entre 2002–2020 [5.]. Veja na Figura 2 um mapa das ocorrências georreferenciadas no GBIF.

**Figura 2.** Mapa interativo das ocorrências georreferenciadas de flebotomíneos da Terra Indígena Wajãpi hospedado pelo GBIF [24.]. https://www.gbif.org/dataset/7140a42f-8ad6-43cd-b252-b5a791c8de4a

### Coleta e processamento dos flebotomíneos

Os flebotomíneos foram coletados com armadilhas de luz semelhantes aos Centros de Controle de Doenças dos EUA (CDC) sem isca, operadas entre 17h e 6h. Armadilhas foram colocadas em áreas utilizadas pelos indígenas, como roças, áreas florestais, áreas de caça, trilhas de acesso e residências.

Os insetos foram armazenados em microtubos contendo álcool 70% e separados dos demais insetos coletados em campo ou sob um microscópio de dissecação em laboratório. Os insetos foram clarificados e montados em lâmina para identificação das espécies, utilizando-se a chave de identificação morfológica de Galati [2.]. Uma subamostra dos insetos será depositada na Coleção de Flebotomíneos do Instituto René Rachou/FIOCRUZ-Minas (COLFLEB/FIOCRUZ).

## Validação de dados e controle de qualidade

Os insetos foram identificados por chaves disponíveis na literatura [2., 3.] por taxonomistas experientes. O conjunto de dados está no formato Darwin Core; 41 termos estão disponíveis para o conjunto de dados Suruwaha e 39 para o conjunto de dados Wajãpi. Todos os campos obrigatórios estão presentes e passaram por triagem no IPT FIOCRUZ. Campos de metadados e conjuntos de dados também estão disponíveis nas páginas online [5., 6.].

## Potential de re-uso dos dados

Esses dados são importantes porque descrevem a distribuição de flebotomíneos coletados em diferentes locais em duas terras indígenas na Amazônia brasileira. Os dados podem ser utilizados por diferentes setores da academia, governo, sociedade civil e organizações não governamentais. No entanto, esses dados podem ser de particular importância para equilibrar o conhecimento científico com o conhecimento indígena para melhorar as atividades de vigilância em saúde e adaptá-las a diferentes contextos ecossociais com a participação dos povos indígenas, que conhecem melhor seus territórios.

Esses dados podem ser usados para enfrentar os desafios no controle das leishmanioses e para entender melhor a epidemiologia dessa doença. Como as medidas de controle no Brasil são baseadas na vigilância e monitoramento de doenças em unidades territoriais, que incluem características biológicas e ambientais, nosso conjunto de dados pode contribuir para uma base de conhecimento mais ampla. Nossos dados fornecem registros de ocorrências de locais que normalmente não seriam pesquisados pelos programas de controle de LTA do sistema público de saúde do governo brasileiro. Esses dados podem ser usados para modelar a distribuição de vetores e doenças no espaço e no tempo, bem como fornecer pistas sobre áreas prioritárias para vigilância e controle de LTA nessas áreas.

Nossos dados de alta qualidade fornecem uma lista validada por especialistas de espécies de flebotomíneos com nomes atualizados de áreas remotas da Amazônia brasileira.

## Data Availability

Os dados que suportam este artigo são publicados através da FIOCRUZ – Fundação Oswaldo Cruz IPT e estão disponíveis sob isenção CC0 do GBIF [21., 24.].

## Nota do editor

Este artigo faz parte de uma série de artigos de Liberação de Dados que trabalham com o GBIF e são apoiados pelo Programa Especial de Pesquisa e Treinamento em Doenças Tropicais (TDR), hospedado na Organização Mundial da Saúde [25.].

## Lista de abreviações

LTA: leishmaniose tegumentar americana; TIS: Terra Indígena Suruwaha; TIW: Terra Indígena Wajãpi.

## Licenças e aprovação ética

As licenças de coleta e alvarás para nossos estudos foram as seguintes: TIS, licença de coleta do SISBIO, emitida pelo Sistema de Autorização e Informação em Biodiversidade (IBAMA) (39337-1) e FUNAI (08620.040969/2013-51); e na TIW, licença de coleta do SISBIO (37935-4), FUNAI (08620.030843/2014-59), aprovação do Comitê de Ética (CONEP - CAAE: 20188213.9.0000.5091), e acesso do IPHAN ao conhecimento tradicional associado para fins de pesquisa científica (01450.008806/ 2014-14).

## Consentimento para publicação

Não aplicável.

## Financiamento

Este projeto foi financiado pelo Conselho Nacional de Desenvolvimento Científico e Tecnológico CNPq (números 474506/2012-6 e 404390/2012-9) – PHFS; Coordenação de Aperfeiçoamento de Pessoal de Nível Superior (Capes) e Fundação de Amparo à Pesquisa do Amapá – Fapeap (004/2013) – ESM.

## Contribuição dos autores

PHFS: provisão de recursos, obtenção de financiamento, conceituação da pesquisa, trabalho de campo, coleta de dados, supervisão, elaboração do manuscrito; DRCA: trabalho de campo, provisão de recursos, coleta de dados; ESM: provisão de recursos, captação de financiamento, conceituação da pesquisa; JACB: preparação de amostras; MPF: trabalho de campo, conceituação da pesquisa, coleta de dados; MEMS: trabalho de campo, provisão de recursos, coleta de dados; MDGGA: preparação da amostra; SFM: preparação da amostra; TSC: preparação da amostra; VRA: preparação e identificação da amostra; LAB: curadoria de dados, revisão do manuscrito.

## References

[1] RangelEF, LainsonR. Flebotomíneos do Brasil. In: RangelEF, LainsonR (eds), Flebotomíneos do Brazil. Rio de Janeiro: Editora Fiocruz, 2003; pp. 23–175.

[2] GalatiEAB. Classification of Phlebotominae. In: RangelEF, LainsonR (eds), Flebotomíneos do Brazil. Rio de Janeiro: Editora Fiocruz, 2003; pp. 23–175.

[3] YoungDG, DuncanMA. Guide to the identification and geographic distribution of Lutzomyia sandflies in Mexico, The West Indies, Central and South America (Diptera: Psychodidae). Mem. Am. Entomol. Inst., 1994; 54: 1–881.

[4] WieczorekJ, BloomD, GuralnickR Darwin core: an evolving community-developed biodiversity data standard. PLoS One, 2012; 7(1): e29715. doi:10.1371/journal.pone.0029715.22238640PMC3253084

[5] Global Biodiversity Information Facility (GBIF) Information Processing Toolkit (IPT). Levantamento de flebotomíneos da Terra Indígena Suruwaha, Sul do Amazonas, Brasil. http://ipt.fiocruz.br/ipt/resource?r=fiocruz_suruwaha_01.

[6] Global Biodiversity Information Facility (GBIF) Information Processing Toolkit (IPT). Levantamento de flebotomíneos da Terra Indígena Wajãpi, Amazônia Oriental, Brasil. http://ipt.fiocruz.br/ipt/resource?r=fiocruz_wajapi_01.

[7] GBIF: The Global Biodiversity Information Facility. What is GBIF? 2022; https://www.gbif.org/what-is-gbif/. Accessed 27 February 2022.

[8] Sistema de Informação de Agravos de Notificação, Ministério da Saúde, Brasil. https://www.gov.br/saude/pt-br/media/pdf/2021/novembro/17/lt-casos.pdf/. Accessed 22 Feb 2022.

[9] CoimbraCEAJr, VenturaSR. Saúde, minorias e desigualdade: algumas teias de inter-relações, com ênfase nos povos indígenas no Brasil. Cien. Saude Colet., 2000; 5(1): 125–132.

[10] AriasJA, FreitasRA. Sobre os vetores da leishmaniose cutânea na Amazônia Central do Brasil. 2. Incidência de Flagelados em flebótomos selváticos. Acta Amazon., 1978; 8: 387–396.

[11] JustinianoSCB, ChagasAC, PessoaFA Comparative biology of two populations of *Lutzomyia umbratilis* (Diptera: Psychodidae) of Central Amazonia, Brazil, under laboratory conditions. Braz. J. Biol., 2004; 64: 227–235.1546229510.1590/s1519-69842004000200007

[12] LainsonR, ShawJJ, ReadyPD XVI. Isolation and identification of Leishmania species from sand flies, wild animals and man in north Pará State, with particular reference to *L. b. guyanenesis* causative agent of ‘pian-bois’. Trans. R. Soc. Trop. Med. Hyg., 1981; 75: 530–536.732412810.1016/0035-9203(81)90192-9

[13] Instituto Brasileiro de Geografia e Estatística. https://cidades.ibge.gov.br/. Accessed 22 February 2022.

[14] GuerraJA, PrestesSR, SilveiraH Mucosal Leishmaniasis caused by *Leishmania* (Viannia) *braziliensis* and *Leishmania (Viannia) guyanensis* in the Brazilian Amazon. PLoS Negl. Trop. Dis., 2011; 8; 5(3): e980. doi:10.1371/journal.pntd.0000980.PMC305090321408116

[15] GrimaldiGJr, MomenH, NaiffRD Characterization and classification of leishmanial parasites from humans, wild mammals, and sand flies in the Amazon region of Brazil. Am. J. Trop. Med. Hyg., 1991; 44(6): 645–661.185896810.4269/ajtmh.1991.44.645

[16] Povos Indígenas do Brasil. https://pib.socioambiental.org/pt/Povo:Zuruah%C3%A3/. Accessed 23 February 2022.

[17] Povos Indígenas do Brasil. https://pib.socioambiental.org/pt/Povo:Waj%C3%A3pi/. Accessed 23 February 2022.

[18] GalloisDT. Terra Indígena Wajãpi: da demarcação às experiências de gestão territorial. São Paulo: Iepé, 2011; http://www.institutoiepe.org.br/media/livros/Livro_Terra_Indigena_Wajapi.pdf.

[19] AparícioM. A vida social e econômica dos Sorowaha. Cuiabá: OPAN - Operação Amazônia Nativa, CIMI - Conselho Indigenista Missionário, Prelazia de Lábrea, 1996.

[20] FankJ, PortaEM. A vida social e econômica dos Sorowaha. Cuiabá: OPAN - Operação Amazônia Nativa, CIMI - Conselho Indigenista Missionário, Prelazia de Lábrea, 1996.

[21] Fernandes ShimabukuroPH. Levantamento de flebotomíneos da Terra Indígena Suruwaha, Sul do Amazonas, Brasil. Version 1.5. FIOCRUZ - Oswaldo Cruz Foundation. Occurrence dataset, 2022; 10.15468/28xvr7.

[22] Plano de Gestão Socioambiental. Terra Indígena Wajãpi como estamos organizados para continuar vivendo bem na nossa terra. Apina, Awatac e Iepé, 2017; https://institutoiepe.org.br/wp-ontent/uploads/2020/07/plano_de_gestao_wajapi.pdf/. Accessed 27 February 2022.

[23] GalloisC. Wajãpi rena: roça, pátios e cassa. 2nd ed., Rio de Janeiro: Museu do Índio/APINA/Iepé, 2009.

[24] Fernandes ShimabukuroPH. Levantamento de flebotomíneos da Terra Indígena Wajãpi, Amazônia Oriental, Brasil. Version 1.3. FIOCRUZ - Oswaldo Cruz Foundation. Occurrence dataset, 2022; 10.15468/gt29ub.

[25] Vectors of human disease series. GigaByte. 2022; 10.46471/GIGABYTE_SERIES_0002.
